# Diverse Presentations of Invasive Aspergillus and Mucorales Infections in Immunocompromised Patients: A Case Series

**DOI:** 10.7759/cureus.92799

**Published:** 2025-09-20

**Authors:** Nidhi Bhatnagar, Nishtha Singh, Ashish Ranjan, Rungmei S K. Marak

**Affiliations:** 1 Microbiology, Hind Institute of Medical Sciences, Lucknow, IND; 2 Urology and Renal Transplantation, Sanjay Gandhi Postgraduate Institute of Medical Sciences, Lucknow, IND; 3 Microbiology, Sanjay Gandhi Postgraduate Institute of Medical Sciences, Lucknow, IND

**Keywords:** aspergillosis, invasive fungal infection, mixed fungal infection, mucormycosis, pulmonary cavity

## Abstract

Invasive fungal infections (IFIs) pose significant challenges in immunocompromised patients, particularly those with diabetes, malignancies, HIV, COVID-19, or prolonged corticosteroid use. Invasive infections due to *Aspergillus* and Mucorales, alone or in combination, are increasingly recognized, often with atypical presentations. These fungal infections can mimic bacterial or tubercular diseases and pose significant therapeutic challenges, as their management requires tailored antifungal regimens, particularly in cases of co-infections.

We present three cases of diverse presentations of *Aspergillus *and Mucorales infections in immunocompromised patients. Case 1: A 65-year-old male with renal and bladder carcinoma developed a cavitary pulmonary lesion; bronchoalveolar lavage grew *Aspergillus niger*, and a concurrent urinary tract infection with *Enterococcus faecium* was identified. Case 2: A 58-year-old diabetic male presented with right-sided rhinosinusitis and visual loss; surgical biopsy demonstrated *Rhizopus arrhizus* and *Aspergillus flavus*. Case 3: A 70-year-old diabetic male with respiratory distress was found to have a hepatic abscess with pleuro-peritoneal extension due to *Rhizopus pusillus*; sputum cultures additionally yielded *Aspergillus fumigatus*.

These cases underscore the evolving clinical spectrum of *Aspergillus *and Mucorales infections. High clinical suspicion, timely imaging, and microbiological confirmation are critical for early diagnosis. Optimal management requires a multidisciplinary approach, combining surgical intervention, targeted antifungal therapy, and control of underlying risk factors. Reporting such rare and atypical presentations contributes to improved awareness and evidence-based strategies in managing these life-threatening infections.

## Introduction

Invasive fungal infections (IFIs) are life-threatening opportunistic diseases that have risen in incidence worldwide, largely due to increasing numbers of immunocompromised patients with uncontrolled diabetes, malignancies, hematological disorders, organ transplantation, or prolonged corticosteroid use [1-3]. Globally, mucormycosis has an estimated incidence ranging from 0.005 to 1.7 per million population, but the prevalence in India is nearly 80 times higher, reflecting the high burden of uncontrolled diabetes and widespread corticosteroid use [3,4]. Aspergillosis, particularly due to *Aspergillus fumigatus*, is another major IFI with diverse pulmonary and extrapulmonary manifestations [2,5]. IFIs due to *Aspergillus* and Mucorales, either individually or concurrently, are being increasingly reported in recent years, often in the backdrop of COVID-19-associated mucormycosis, although they remain underreported and underdiagnosed [4,6].

The pathology of these infections is characterized by angioinvasion, thrombosis, and tissue necrosis, which account for their aggressive course and high mortality [1,7]. Pulmonary involvement may present as nodules, cavitary lesions, or consolidations that can closely mimic bacterial pneumonia or pulmonary tuberculosis, leading to diagnostic delays [5,8]. Similarly, rhino-orbito-cerebral disease due to Mucorales may coexist with *Aspergillus* spp., creating overlapping clinical and radiological features that complicate recognition [6]. Early and accurate diagnosis is further limited by the low sensitivity of cultures, difficulties in distinguishing colonization from invasive disease, and the lack of reliable biomarkers for Mucorales [1,7].

From a therapeutic standpoint, treatment requires prompt initiation of antifungal therapy and, where feasible, surgical debridement. Amphotericin B is the first-line drug for mucormycosis, whereas voriconazole remains the drug of choice for aspergillosis; isavuconazole is an accepted alternative in selected cases [1,2]. However, management of dual infections is particularly challenging, as there are few antifungals active against both groups, and combination or sequential therapy is often necessary [7]. Prognosis remains guarded, with reported mortality rates exceeding 50% in disseminated or delayed-diagnosis cases [3,4].

Given these challenges, documenting unusual presentations of *Aspergillus* and Mucorales infections, alone or in combination, is crucial to increasing clinical awareness, guiding early diagnosis, and informing therapeutic strategies. Herein, we present three distinct cases presented to our department between February 2021 and July 2021 of invasive *Aspergillus* and *Aspergillus*-Mucorales coinfections in immunocompromised patients, including a rare hepatopulmonary fistulizing manifestation, highlighting the diagnostic dilemmas and management complexities associated with these emerging infections.

## Case presentation

Case 1

**Figure 1:**
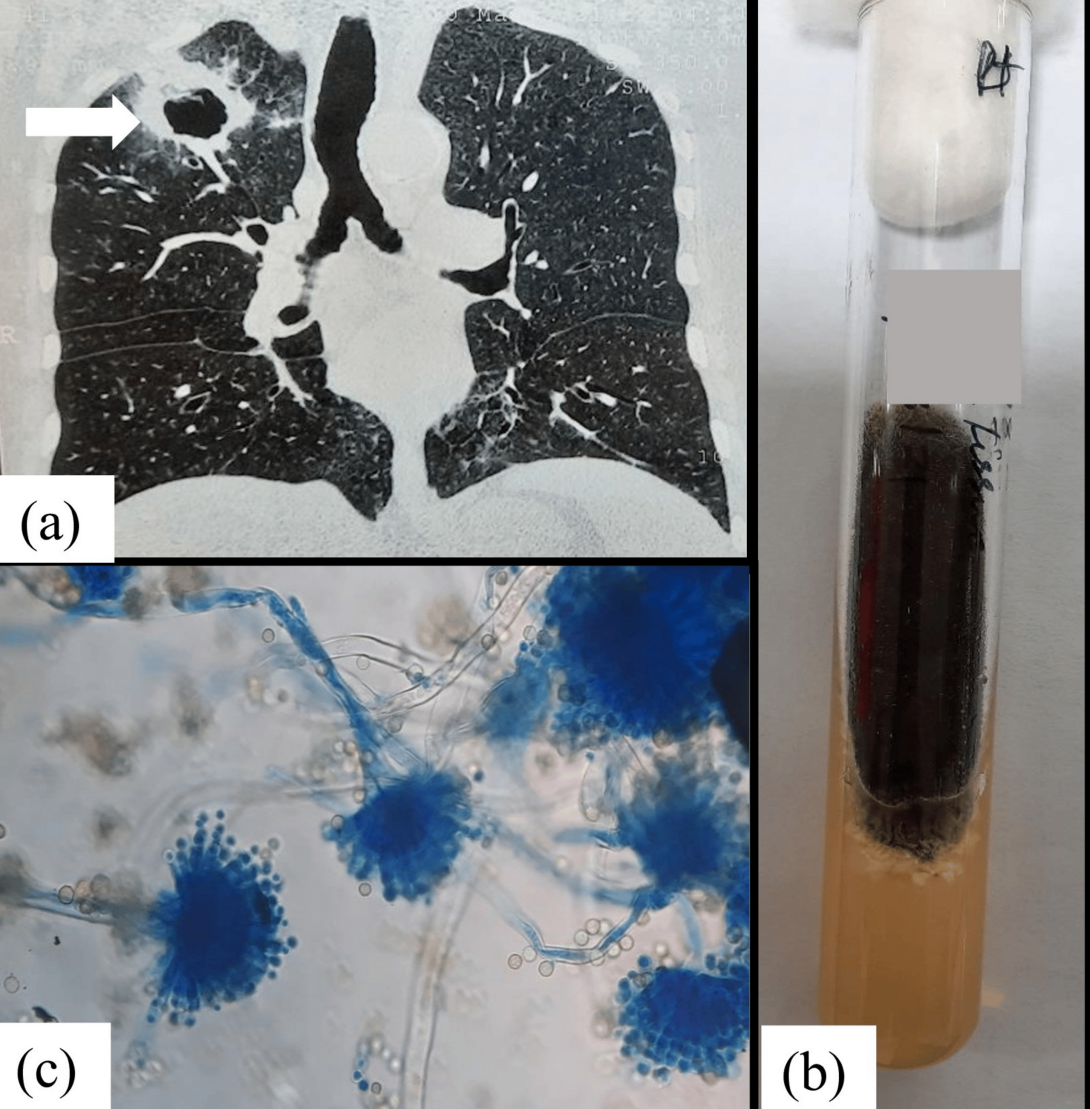
Case 1: (a) High-resolution computed tomography (HRCT) of the thorax showing a thick-walled cavity with surrounding ground-glass densities in the right upper lobe (arrow). (b) Culture of lung tissue on SDA showing black cottony growth of Aspergillus niger. (c) Microscopic appearance of a lactophenol cotton blue mount (40× magnification) from the SDA culture showing long, smooth, unbranched conidiophores terminating in flask-shaped phialides covering the entire vesicle, characteristic of A. niger. SDA: Sabouraud dextrose agar

A 65-year-old male, a known case of left renal cell carcinoma with bladder carcinoma, presented with cough and expectoration of two weeks’ duration. Chest imaging revealed a cavitary lesion in the lung. Bronchoalveolar lavage (BAL) fluid was subjected to microbiological examination, which demonstrated septate hyphae and subsequently grew *Aspergillus niger* on culture (Figure 1).

During hospitalization, the patient also developed urinary symptoms, and urine culture confirmed *Enterococcus faecium.* He was managed with intravenous liposomal amphotericin B followed by oral voriconazole for aspergillosis, along with appropriate antibiotics for the urinary tract infection, in addition to supportive care. Antifungal therapy led to symptomatic improvement with a reduction in cavity size.

Case 2

**Figure 2:**
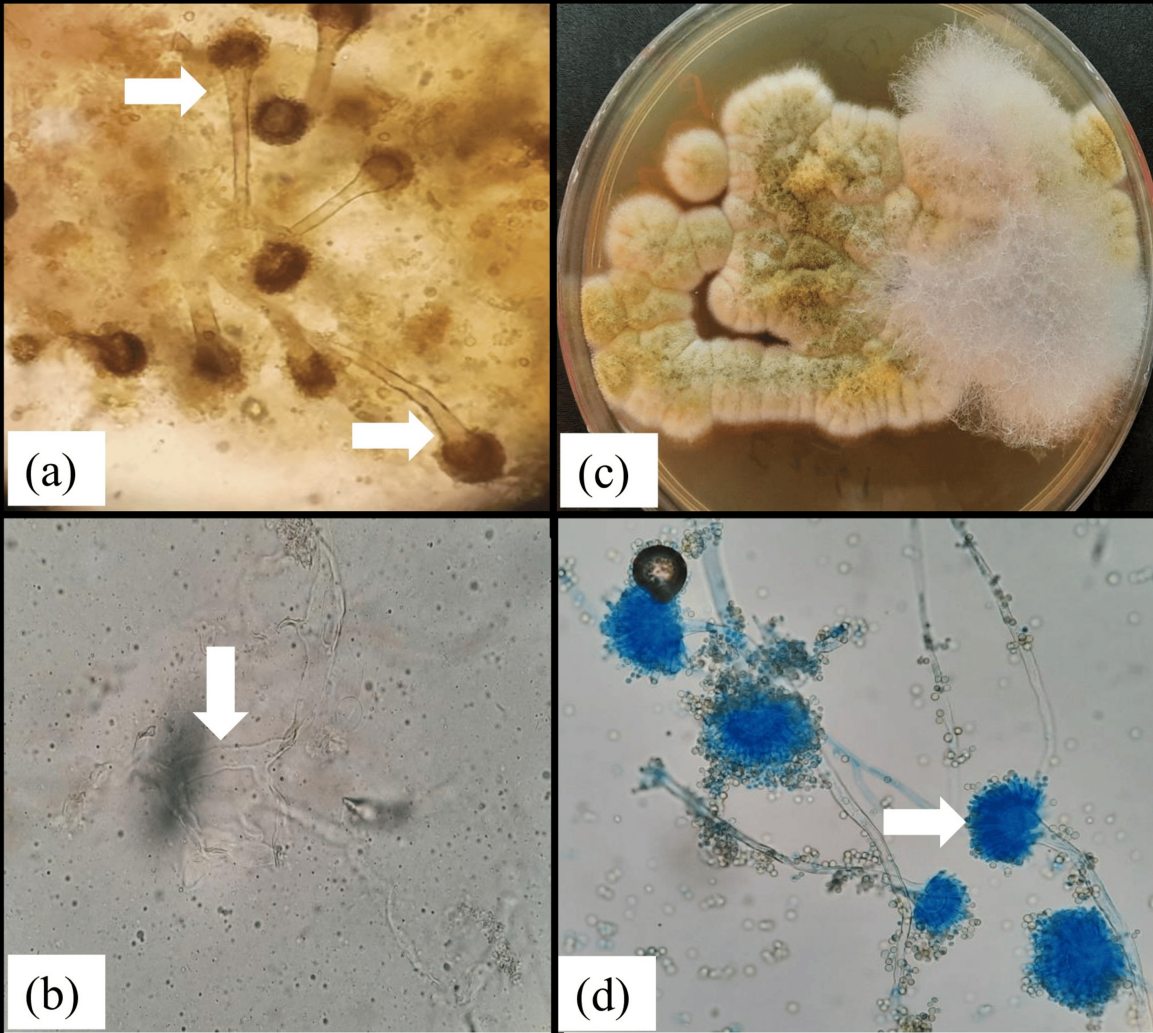
Case 2: (a) Microscopic image of a KOH wet mount (40× magnification) of nasal tissue obtained by FESS showing numerous conidial heads of Aspergillus spp. (arrow). (b) Broad, ribbon-like aseptate hyphae with right-angled branching, suggestive of Mucormycetes (downward arrow). (c) Culture of nasal tissue on SDA showing yellowish-green woolly growth of Aspergillus flavus and white cottony growth of Mucormycetes. (d) Microscopic appearance of a lactophenol cotton blue mount (40× magnification) of yellowish-green mycelial growth from the SDA plate showing long, unbranched conidiophores terminating in globose conidia and uniseriate phialides covering the entire vesicle, characteristic of A. flavus (arrow). KOH: potassium hydroxide; FESS: functional endoscopic sinus surgery; SDA: Sabouraud dextrose agar

A 58-year-old diabetic male presented with fever, right-sided headache, nasal discharge, and progressive diminution of vision in the right eye. Computed tomography (CT) of the paranasal sinuses revealed pansinusitis with evidence of invasive fungal disease. The patient underwent functional endoscopic sinus surgery (FESS), and tissue samples were sent for microbiological and histopathological evaluation. Direct microscopy demonstrated broad, aseptate hyphae as well as septate hyphae with acute-angle branching. Cultures confirmed the presence of *Rhizopus arrhizus* and *Aspergillus flavus* (Figure 2).

Histopathology corroborated invasive fungal rhinosinusitis [6]. The patient was treated with surgical debridement, followed by antifungal therapy with liposomal amphotericin B, along with strict glycemic control and supportive measures. Clinical improvement was observed with this combined approach.

Case 3

**Figure 3:**
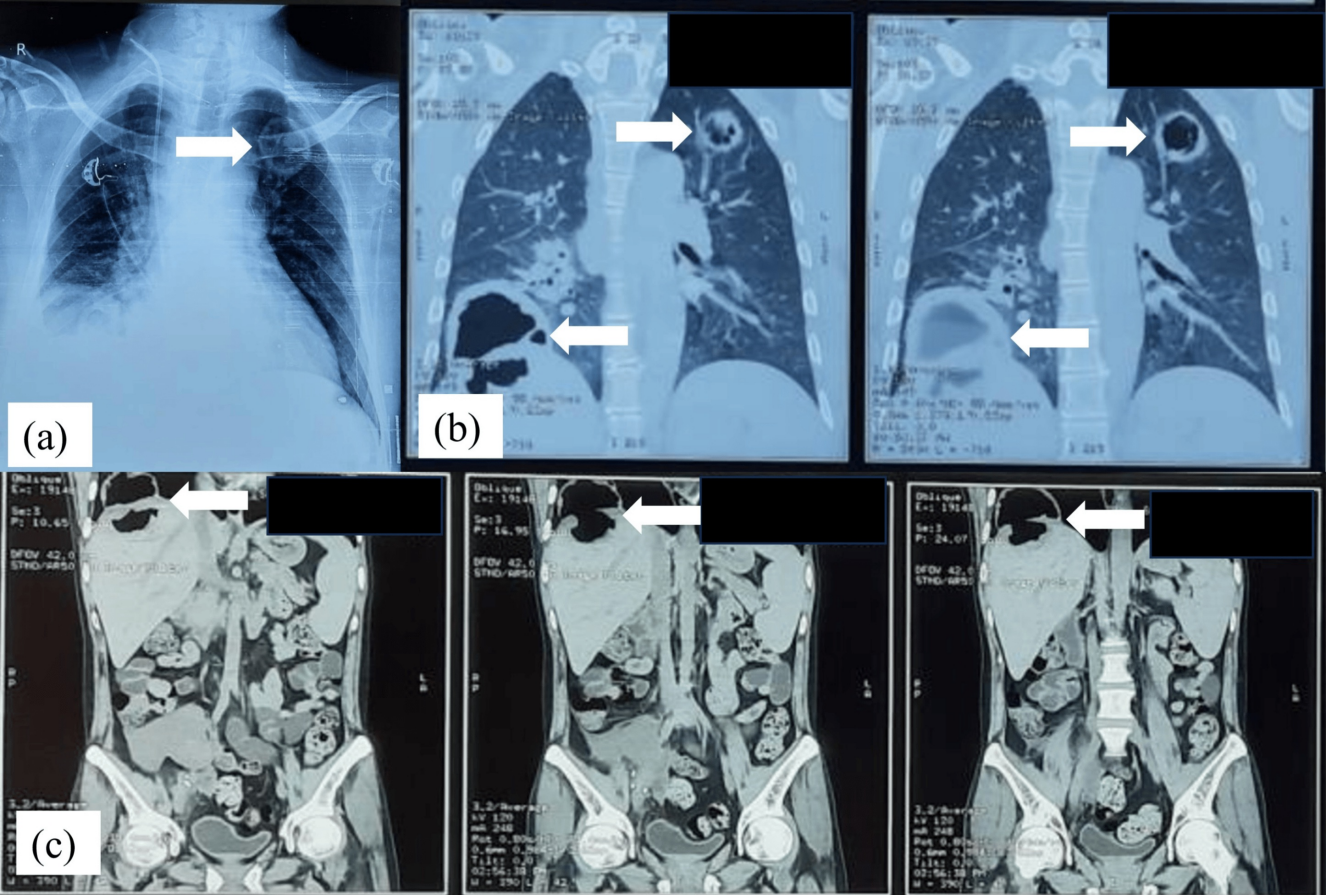
Case 3: (a) Chest X-ray showing a cavitary lesion in the upper lobe of the left lung (arrow). (b) High-resolution computed tomography (HRCT) of the thorax showing a thick-walled necrotic lesion in the left upper lobe (right arrow) and a thick-walled hypodense lesion in the right lobe of the liver extending through the diaphragm into the lower lobe of the right lung (left arrow). (c) CT scan of the abdomen showing a thick-walled hypodense lesion in hepatic segments VII/VIII extending into the lower lobe of the right lung through a defect in the right dome of the diaphragm (arrow).

A 70-year-old male with long-standing diabetes mellitus presented with fever, cough, and respiratory distress. Imaging studies revealed a cavitary lesion in the right lower lobe of the lung with communication from a hepatic abscess, suggestive of pleuro-peritoneal extension. Microbiological examination of aspirated hepatic material showed broad, aseptate hyphae, and culture confirmed *Rhizopus pusillus*. Sputum examination, however, revealed both broad, aseptate hyphae and septate hyphae with acute-angle branching, and cultures grew *R. pusillus* along with *Aspergillus fumigatus* (Figure 3).

The patient was managed with antifungal therapy with injection liposomal amphotericin B and oral posaconazole, appropriate antibiotics, and supportive measures. An ultrasound-guided percutaneous drainage catheter was inserted and left in situ for the liver abscess drainage. Multidisciplinary care was instituted, given the complex presentation involving both hepatic and pulmonary structures. On follow-up, ultrasound showed a residual hepatic cavity with intralesional air suggestive of communication with the lung cavity. The patient was stabilized on antifungal therapy, discharged, and subsequently underwent surgical debridement at another institute.

Table 1 summarizes all three cases.

**Table 1:** Clinical characteristics, microbiological findings, management, and outcomes of three patients with invasive Aspergillus and/or Mucorales infections. BAL: bronchoalveolar lavage; FESS: functional endoscopic sinus surgery

Case	Age/sex	Risk factors	Site of involvement	Organisms isolated	Management	Outcome
1	65/M	Renal cell carcinoma, bladder carcinoma	Pulmonary cavity	*Aspergillus niger *(BAL); *Enterococcus faecium* (urine)	Liposomal amphotericin B → Oral voriconazole + antibiotics + supportive care	Recovery
2	58/M	Diabetes mellitus	Rhino-sinus with orbital involvement	*Rhizopus arrhizus* and *Aspergillus flavus* (FESS biopsy)	Surgical debridement + liposomal amphotericin B → Oral voriconazole + glycemic control	Recovery
3	70/M	Diabetes mellitus	Hepatic abscess with pleuro-pulmonary extension	*Rhizopus pusillus* (hepatic abscess); *R. pusillus* + *Aspergillus fumigatus* (sputum)	Oral posoconazole + antibiotics + Percutaneous drainage tube insertion + multidisciplinary care	Stabilized (guarded prognosis)

## Discussion

IFIs due to *Aspergillus* and Mucorales, either individually or concurrently, are being increasingly reported, particularly in immunocompromised patients [3,4]. The predisposing risk factors include diabetes mellitus, hematological malignancies, organ transplantation, HIV, COVID-19, and prolonged corticosteroid use [1,2,6]. Similar to earlier studies, diabetes mellitus and malignancy were the major risk factors in our series, suggesting that the changing epidemiology of immunosuppression continues to shape disease burden.

Pulmonary cavities due to *A. niger* are uncommon, yet they can cause serious infection in immunocompromised hosts, unlike the more frequent *A. fumigatus* reported in larger series [5]. Similarly, dual infections of rhinosinusitis by *Rhizopus* and *Aspergillus* spp. complicate clinical recognition, given their overlapping features with bacterial sinusitis or pulmonary tuberculosis [7]. Our case of concurrent *Rhizopus* and *A. flavus* infection underscores the need to consider mixed fungal etiologies when conventional therapy fails.

The third case in our series is particularly remarkable because of the rare presentation of a hepatic abscess breaching into the pleuro-peritoneal cavity and communicating with a pulmonary cavity. In this patient, the liver abscess was caused by *R. pusillus*, while sputum cultures demonstrated both *R. pusillus* and *A. fumigatus*. Such hepatopleural or hepato-bronchial fistulae are rarely described in the literature, and even more unusual is their association with mixed fungal infections [8,9]. Most published cases of hepatic abscess fistulization have bacterial etiologies, and even in fungal infections, *Candida* has been more commonly implicated than Mucorales [10]. The unique mixed fungal involvement in our patient highlights the aggressive tissue invasiveness of Mucorales, known for angioinvasion and necrosis.

The clinical implications of this rare complication are significant. First, the presentation may mimic empyema, complicated pneumonia, or pyogenic liver abscess rupture, thereby delaying diagnosis. This mirrors earlier reports where delayed recognition of fungal etiology contributed to poor outcomes [8]. Second, management requires close coordination between hepatobiliary surgery, pulmonology, infectious diseases, and microbiology teams. In our patient, early recognition and institution of antifungal therapy, along with supportive measures, likely contributed to stabilization. However, prognosis in such cases remains guarded, especially when the diagnosis is delayed.

From a therapeutic perspective, amphotericin B continues to be the first-line therapy for mucormycosis [1], while triazoles such as voriconazole or isavuconazole are preferred for aspergillosis [2]. In mixed infections, sequential or combination therapy is generally required, as also reported in earlier studies of concurrent aspergillosis and mucormycosis, where dual antifungal regimens improved outcomes compared to monotherapy [11,12]. Surgical intervention remains essential where feasible, particularly in rhino-orbital disease or localized pulmonary lesions, as debridement reduces fungal burden and improves antifungal penetration.

Overall, this case series underscores three important messages: (1) *Aspergillus* and Mucorales infections, alone or in combination, are increasingly recognized in immunocompromised hosts and may present with atypical manifestations; (2) cavitary disease due to *A. niger* and dual rhinosinusitis with *Rhizopus* and *A. flavus* are diagnostic challenges requiring combined microbiological and radiological input; and (3) the rare occurrence of hepatopleural fistula with pulmonary cavity in the third case demonstrates the aggressive potential of these pathogens and highlights the need for early multidisciplinary intervention.

## Conclusions

Invasive *Aspergillus*-Mucorales infections, alone or in combination, are increasingly recognized among immunocompromised patients such as those with diabetes, malignancies, or prior corticosteroid use. These infections often mimic bacterial pneumonia, sinusitis, or tuberculosis, which contributes to delays in diagnosis and worsens prognosis. Our case series highlights the spectrum of clinical presentations, ranging from pulmonary cavities due to *A. niger* to dual fungal rhinosinusitis and the rare occurrence of a hepatic abscess fistulizing into the pleuro-pulmonary cavity.

Early recognition of such infections requires a high index of suspicion, multidisciplinary input, and reliance on both microbiological and radiological evidence. Prompt initiation of antifungal therapy, judicious use of surgical interventions, and strict control of underlying risk factors remain essential for improving patient outcomes. Continued reporting of such unusual manifestations is important to strengthen global understanding of IFIs, guide timely diagnosis, and refine therapeutic strategies in this challenging clinical domain.
